# Acute coronary syndromes in chronic hemodialysis patients: a series of 34 cases (case series)

**DOI:** 10.1097/MS9.0000000000000941

**Published:** 2023-06-08

**Authors:** Mohammed Boutaybi, Badia Aloutmani, Mohammed El-Azrak, Nabila Ismaili, Noha El Ouafi

**Affiliations:** a Department of Cardiology, Mohammed VI University Hospital; b Epidemiological Laboratory of Clinical Research and Public Health, Faculty of Medicine and Pharmacy of Oujda, Mohammed First University, Oujda, Morocco

**Keywords:** calcifications, case series, chronic hemodialysis, coronary artery bypass graft, triple vessel diseases

## Abstract

Cardiovascular diseases, particularly acute coronary syndrome, are the leading cause of death in chronic hemodialysis patients. Our study aims to analyze the pathophysiological, clinical, angiographic, and therapeutic characteristics of coronary heart disease in hemodialysis patients.

**Patients and Methods:**

This single-centered retrospective descriptive study included 34 hemodialysis patients hospitalized in the cardiovascular ICU.

**Results:**

The mean age of patients in our study was 64.4±11.3 years. The main cardiovascular risk factor found in our study was age, with a prevalence of 76.50%, followed by hypertension, with a prevalence of 67.60%. Diabetes was present in 55.90% of patients. The authors also found that 17.90% of patients were obese, and 29.40% had abdominal obesity. The main cause of renal disease in our study was diabetic nephropathy (52.90% of cases), followed by hypertensive nephropathy (23.50% of cases). ST segment elevation myocardial infarction was found in 14.70% of cases, and non-ST-segment elevation myocardial infarction in 85.30% of cases. Coronary angiography was performed in 76.40% of patients. Single-vessel coronary artery disease (CAD) was found in 20%, two-vessel CAD in 50%, and three-vessel CAD in 30% of the cases. Coronary artery calcifications were observed in 21.42% of cases. 38.23% had an angioplasty, and 20.58% were referred for a coronary artery bypass graft.

**Conclusion:**

Despite the high mortality rate after acute coronary syndrome, hemodialysis patients are less likely to undergo diagnostic angiography or coronary revascularization. Patients on hemodialysis tend to have multiple, diffuse, calcified CAD.

## Introduction

Highlights

- Cardiovascular disease is the leading cause of death in dialysis patients.
- Hemodialysis patients were less likely to undergo coronary angiography.
- Hemodialysis patients have multiple, diffuse, calcified coronary artery lesions.
- Long-term survival after bypass surgery is better than coronary angioplasty.

Chronic kidney disease (CKD) is a global public health problem. The size of the global population with CKD and end-stage renal disease continues to increase due to the high prevalence of cardiovascular risk factors.

Cardiovascular disease is the leading cause of death in hemodialysis patients, accounting for ~40% of all deaths, and ~17% of deaths are attributable to acute coronary syndromes (ACS)^1^.

Hemodialysis patients are at increased risk for coronary artery disease, as a combination of classic cardiovascular risk factors in addition to uremia-specific risk factors accelerates the progression of atherosclerosis, explaining the increased incidence of cardiovascular events^2,3^.

ACS in chronic hemodialysis patients present unique features regarding pathophysiological, diagnostic, angiographic, and therapeutic aspects.

Our study aims to investigate the clinical, pathophysiological, angiographic, and therapeutic features of ACS in hemodialysis patients and compare our findings to those reported in the literature.

## Patients and methods

It is a single-centered retrospective descriptive study conducted over a period of 7 years from January 2016 to December 2022. We included 34 hemodialysis patients hospitalized in the cardiovascular ICU during the initial phase of acute coronary syndrome with or without persistent ST segment elevation.

Data collection was performed according to the United States National Cardiovascular Data Registry (NCDR) Cath-PCI Registry^4^.

Age is considered a cardiovascular risk factor when it is greater than 50 years for men and greater than 60 years for women.

Patients were considered hypertensive: when frequent measurements of systolic blood pressure and/or diastolic blood pressure greater than or equal to 140/90 mmHg were performed throughout the hospitalization, or when they are known to have hypertension and are on antihypertensive treatment.

Diabetes was determined on the basis of the following criteria: previously known diabetic patients using antidiabetic treatments, or with fasting blood glucose greater than or equal to 1.26 g/l in two blood tests, glycated hemoglobin HBA1C greater than or equal to 6.5%.

Obesity is defined by a BMI (weight/height^2^) greater than 30 kg/m^2^. Abdominal obesity is defined by a waist circumference greater than 94 cm in men or greater than 80 cm in women.

Smoking was defined as a cardiovascular risk factor in patients who smoked 1 or more cigarettes per day in the past 3 years^5^.

Normal values for biological results are expressed in (Table 2).

The ACS were classified as follows:

Non-ST-segment elevation myocardial infarction (NSTEMI), determined by the presence of characteristic symptoms of myocardial ischemia and ST-segment depression, an inverted T wave or normal ECG, and positive cardiac enzymes.

ST-segment elevation myocardial infarction (STEMI), defined by the presence of characteristic symptoms of myocardial ischemia in association with persistent ECG ST elevation and the release of biomarkers of myocardial necrosis^6^.

The Killip classification was used to assess left ventricular (LV) performance in patients at admission^7^.

The severity of dyspnea was defined using the New York Heart Association heart failure classification^8^.

Transthoracic echocardiography was performed in all patients using the 4D cardiovascular ultrasound apparatus. The probe used was the GE M5SC-D cardiac probe. LV performance was assessed according to the ejection fraction, calculated according to the Simpson method. Patients are classified into three groups according to left ventricular ejection fraction (LVEF), preserved EF for an EF greater than or equal to 50%, moderately impaired EF for an EF between 49 and 41%, impaired EF for an EF less than or equal to 40%^9^.

The syntax score is calculated in patients with three-vessel coronary artery disease or left main coronary to assess the complexity of the coronary artery disease and to aid in decision-making regarding revascularization strategy. Scores are divided into tertiles ((0–22). (23–32). (>32)).

This study included all hemodialysis patients hospitalized during the acute phase of STEMI and NSTEMI. Nondialysis dependent patients and those with postinfarction beyond 24 h and those with previous ECG changes consistent with myocardial ischemia but without new evidence of acute coronary disease were excluded.

The retrospective analysis was done by collecting data from computerized medical records on the electronic information system.

Informed consent was waived due to the study’s retrospective design, based on our institutional policies for de-identified case series.

This study was performed in compliance with the standards and principles set forth by the Preferred Reporting of Case Series in Surgery (PROCESS) guidelines^10^.

The data were entered and analyzed using SPSS 20.0.1 software. The Kolmogorov–Smirnov test was applied to determine the distribution of the data. A *P* value<0.05 was considered statistically significant. The mean and SD were used for normally distributed variables, and the median and interquartile range were used for variables not normally distributed, while the categorical variables were presented as frequencies and percentages.

## Results

From January 2016 to December 2022, we collected 2684 patients with acute coronary syndrome with or without ST-segment elevation, of which 34 cases were chronic hemodialysis patients representing 1.26% of cases. Fourteen were women and 20 were men, for a sex ratio of 0.7 (F/M). Of the 34 patients, 14.70% were STEMI, and 85.30% were NSTEMI. The mean age in our study was 64.4±11.3 years, with a minimum of 37 years and a maximum of 88 years. The main cause of renal disease in our study was diabetic nephropathy (52.90% of cases), followed by hypertensive nephropathy (23.50% of cases). Obstructive nephropathy was found in 5.90% of cases, polycystic kidney disease was found in 5.90% of cases, and the etiology remained undetermined in 11.80% of cases. The average duration of hemodialysis was 5.4±0.6 years, and the average number of sessions was 2.3 sessions/week.

**Figure 1:**
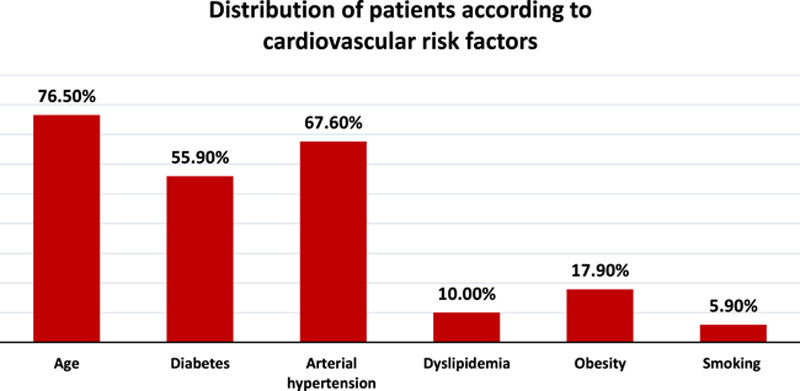
Distribution of patients according to cardiovascular risk factors.

Age was the most common cardiovascular risk factor identified in our study, with a prevalence of 76.50% of cases, followed by hypertension, with a prevalence of 67.60%. Diabetes was present in 55.90% of cases. On average, the duration of diabetes in these patients was 8.3±2.3 years. In our study, 17.90% of patients were obese, and 29.40% had abdominal obesity. A known dyslipidemia was found in 10% of our patients. The least represented risk factor in our study was smoking, with a prevalence of 5.90% (Fig. 1).

In our study, 28.50% of patients presented with grade III–IV dyspnea. Typical chest pain was noted in 79.40% of cases, with epigastralgia in 11.90%. Associated symptoms of nausea and vomiting were present in 38% of the patients in our study.

**Figure 2:**
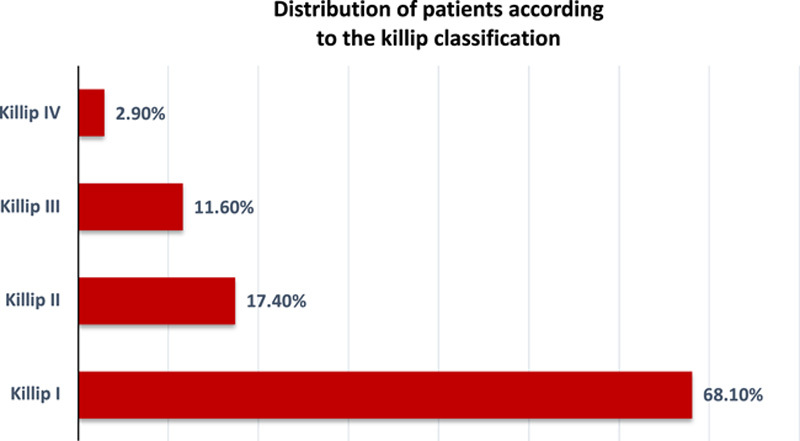
Distribution of patients according to the Killip classification.

The average blood pressure was 136/73 mmHg, with extreme values ranging from 80 to 190 mmHg for systolic blood pressure and 50–100 mmHg for diastolic blood pressure. The average heart rate was 80±14 bpm, with extreme values ranging from 42 to 140 bpm. Out of all the patients, 29% exhibited symptoms of heart failure, the Killip classification of the patients (Fig. 2). A mitral regurgitation murmur was found in 2.90% of cases, and a bruit originating in major vascular areas was noted in 2.90% of cases.

We performed a synthesized 18-lead ECG for all patients upon their admission to the emergency department and then again in the cardiac ICU. The rhythm was sinus in 88.20% of cases, 8.82% had atrial fibrillation, and 2.90% had an electro-paced rhythm. ST segment elevation was present in 14.70% of cases, 58.80% presented with ST segment depression, and 52.90% had left ventricular hypertrophy.

In our study, LVEF ranged from 25 and 70% with a median of 49±12.7%. LV systolic dysfunction was found in 52.90% of cases, with severe dysfunction (LVEF≤40%) in 26.47% of cases. 64.70% had LV hypertrophy. Ventricular akinesia was observed in 52.90% of cases and hypokinesia in 26.50% of cases. 23.50% of patients presented with low to moderate pericardial effusion without signs of tamponade. The other findings are described in (Table 1).

**Table 1:** Distribution of patients by echocardiographic abnormalities.

Echocardiographic abnormalities	Prevalence (%)
LV dilation	2.90%
RV Dilation	8.80%
Mild MR	29.40%
Akinesia	52.90%
Hypokinesia	26.50%
Thrombus	2.90%
LVH	64.70%
Pericardial effusion	23.50%
Pulmonary Arterial Hypertension	29.40%

The average of the biological results is expressed in (Table 2).

**Table 2:** The average of the biological results.

Examen	Results	Normal values
Hemoglobin (g/dl)	9.76±0.38	Men 13–17 Women 12–16
Platlets (E/mm^3^)	233735±17610	(150 000–400 000)
White blood cells (E/mm^3^)	12349±2501	(4000–10 000)
Creatinine (mg/l)	87.47±5.4	(6–12)
Calcium (mg/l)	87.08±1.9	(90–105)
Phosphorus (mg/l)	59.86±17.82	(30–45)
Parathormone (pg/ml)	301±221	(0–65)
Ferritin (ng/ml)	687±298	Men 30–300 Women 30–200
HbA1c %	6.61±1.48	<7
LDL (g/l)	0.9±0.05	<0.55
HDL (g/l)	0.36±0.06	>0.40
TG (g/l)	1.48±0.16	<1.50
Uric acid (mg/l)	67.38±3.3	(25–60)
Troponin (ng/l)	23472.60±11816.36	<26
C-reactive protein (mg/l)	48.65±11.35	<6

Coronary angiography was performed in 76.40% of patients, mainly with a radial approach (73.50%). Right dominance was present in 70.60% of cases. The prevalence of left and balanced dominance were 11.80 and 17.60%, respectively. The coronary angiography turned out normal in 17.85% of cases, tight stenoses were found in 71.42% of cases, and in 10.71% of cases no significant lesions were found (Table 3).

**Table 3:** Distribution of patients according to coronary angiography data.

Angiography data	Pourcentage (%)
Coronary angiographies	76.40
tight stenoses	71.42
Atheroma without significant lesion	10.71
Normal coronary angiography	17.85

**Figure 3:**
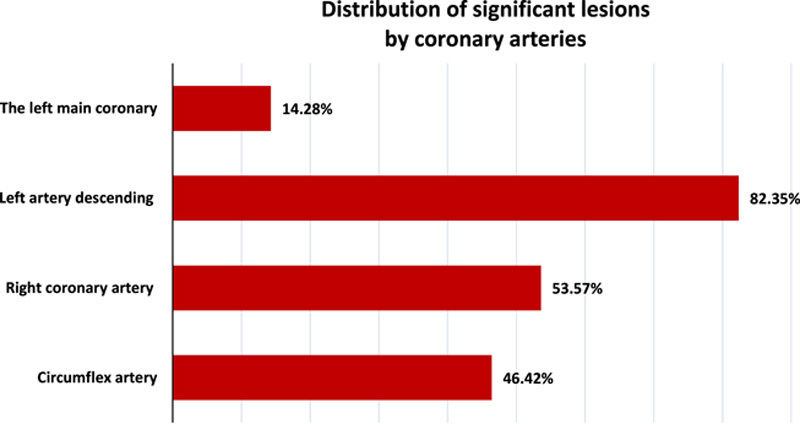
Distribution of significant lesions by coronary arteries.

In our study, single-vessel coronary artery disease (CAD) was found in 20%, two-vessel CAD in 50%, and three-vessel CAD in 30% of the cases. Involvement of the left artery descending (LAD) was found in 82.35% of cases. Involvement of the circumflex artery and the right coronary artery was observed in 46.42 and 53.57% of cases, respectively. Significant left main CAD was observed in 14.28% of cases (Fig. 3). Coronary artery calcifications were observed in 21.42% of our patients. The average syntax score was 28.4±1.2. In 21.52% of patients the score is less than or equal to 22, in 49.82% of cases the score is between 23–32, and in 28.66% of patients the score is greater than or equal to 33.

Coronary angioplasty by active stents was performed in 38.23% of cases. The length of the stents varies between 12 mm and 34 mm with a diameter between 2.25 mm and 3.5 mm. A successful angioplasty was defined as the restoration of TIMI III flow in 92.30% of cases. Coronary artery bypass grafting (CABG) was indicated in 20.58% of cases, myocardial viability testing before coronary revascularization was indicated in 2.90% of cases, and medical treatment was recommended in 32.35% of cases.

In-hospital complications were observed in 46.40% of our study, mainly left heart failure (29% of cases), followed by a ventricular rhythm disorder such as ventricular tachycardia (5.80% of cases). Cardiogenic shock was observed in 2.90% of cases, high-grade conduction disorder in only one patient, and 5.80% of patients died.

## Discussion

According to data from the United States Renal Data System (USRDS), cardiovascular diseases are the leading cause of death among hemodialysis patients, accounting for ~40% of all deaths, and about 17% of deaths can be attributed to ACS^1^.

The Belding Scribner group proposed the term accelerated atherosclerosis to describe the abnormally high mortality in hemodialysis patients compared with subjects of the same age and sex in the general population. Lindner *et al*. had assumed that hemodialysis promotes the accelerated development of atherosclerosis. The incidence of myocardial infarction observed in these patients in the Framingham study was 10 times at an equal age than in the hypertensive population^11^. Therefore, traditional cardiovascular risk factors (diabetes, hypertension, dyslipidemia, and smoking) cannot explain the increased incidence of cardiac death in hemodialysis patients, and nontraditional risk factors such as inflammation, anemia, endothelial dysfunction, and altered calcium phosphate metabolism are associated with atherosclerosis in acceleration-related hemodialysis patients^11^.

Myocardial ischemia in hemodialysis patients can occur in the absence of coronary stenosis. It can be related to an imbalance between myocardial oxygen supply and demand. A decrease in oxygen supply predisposes to arterial hypotension, anemia, and an increase in LV end-diastolic pressure during dialysis. An increase in myocardial oxygen demand may be secondary to LV hypertrophy, hypertension, or tachycardia. Numerous studies have shown that chronic renal failure is associated with abnormal coronary microcirculation, leading to myocardial ischemia through organic modifications (increase in intramyocardial arteriolar wall thickness) and functional modifications (abnormal production of endothelin and NO)^12^.

The atypical presentation of ACS in chronic hemodialysis patients has been demonstrated in numerous studies^13,14^. Sosnov *et al*.^13^ reported that hemodialysis patients who had a myocardial infarction were significantly less likely to report typical chest pain. According to Shroff *et al*.^15^, chest pain was reported as the first symptom in only 40% of hemodialysis patients who had a myocardial infarction, while dyspnea was the most common symptom in the majority of these cases. This atypical clinical presentation of ACS in hemodialysis patients may be explained by uremic neuropathy in addition to diabetic neuropathy in patients who have diabetes as well^16^.

ECG results compatible with ischemia are not typical in hemodialysis patients, as these subjects generally present with left ventricular hypertrophy or left bundle branch block. According to a US study, ST segment elevation was found in only 15.90% of patients with advanced CKD presenting with ACS, compared with 32.50% of patients without CKD. In our study, we found that 85.30% of cases were NSTEMI and 14.70% a STEMI, which is consistent with the results of the GRACE registry showing that ACS without ST-segment elevation is the most common presentation of ACS in hemodialysis patients^14^.

In patients with CKD, the specificity of troponin is reduced by its chronic elevation, which is related to ‘subclinical’ myocardial injury. Hemodialysis patients may have an increased risk to develop silent micro-infarcts^17^, for which atherosclerotic disease is not the only explanation. Indeed, arteriosclerosis, intima-media hypertrophy, and uremic pericarditis can also lead to a reduction in coronary blood flow, leading to minor ischemic lesions^18^.

Hemodialysis patients were less likely to undergo coronary angiography. According to an American study of 154 692 patients admitted for acute myocardial infarction, dialysis patients, and nondialysis dependent CKD patients were significantly less likely to undergo coronary angiography than patients with normal kidney function (42 vs. 45 vs. 56%)^19^.

In our study, coronary angiography was performed in 76.40% of the cases. Hemodialysis patients often have multiple, diffuse, calcified coronary artery lesions.

In our study, single-vessel CAD was found in 20%, two-vessel CAD in 50%, and three-vessel CAD in 30% of the cases. LAD involvement was the most frequent coronary lesion and was found in 82.35%. Similar results were found in a study from Romania in which 27% of the cases had three-vessel, and the most frequent coronary lesion was LAD. The distribution of coronary lesions in this study showed that the proximal segments of the coronary arteries such as the LAD, the circumflex artery, and the right coronary artery, have a higher incidence than the other segments^20^.

In our study, coronary angioplasty was performed in 38.23% of cases. In chronic hemodialysis patients, coronary angioplasty with stenting is performed with a very high immediate success rate, comparable to the results obtained in patients without renal failure^21^. In contrast, the risk of in-hospital cardiac events remains higher (6–10% in dialysis patients versus 2% in patients without renal failure), with a high rate of restenosis and revascularization^22,23^. This restenosis rate is even higher in diabetic patients^24^.

Early invasive therapy after ACS and coronary artery bypass graft surgery reduces mortality in hemodialysis patients^25,26^. Studies have shown that coronary revascularization after myocardial infarction in dialysis patients significantly improves survival compared to medical therapy alone^27^.

The optimal revascularization strategy in hemodialysis patients remains to be determined. In a large retrospective study of a national US database, 7419 end-stage renal disease patients who underwent coronary artery bypass graft surgery were compared with 6887 end-stage renal disease patients who underwent percutaneous coronary intervention. Helgog *et al*.^28^ showed higher perioperative mortality in patients treated with CABG surgery (12.50% vs 5.40%); however, this rate was similar at 1 year and significantly lower in the PCI-treated group after 2 years.

Comparing different strategies of coronary revascularization; long-term survival after CABG surgery is superior to percutaneous coronary intervention in hemodialysis patients. However, the choice between angioplasty and bypass depends on the anatomy of the coronary lesion, surgical risk, and associated lesions. Coronary angioplasty is the preferred treatment for high-risk, multivessel patients with accessible culprit lesions for stenting. Bypass is preferred for patients with multivessel diseases, left main coronary artery involvement, and acceptable surgical risk, or associated valve surgery^29,30^.

### Limitations of the study

This is a descriptive study of the current situation of hemodialysis patients with acute coronary syndrome in the eastern region of Morocco. It does not allow for the analysis of correlations and cause and effect relationships.

It is not comparative with nondialysis dependent patients during the study period.

## Conclusion

Cardiovascular diseases are the leading cause of death in hemodialysis patients. Despite the high mortality rate after ACS, dialysis patients are significantly less likely to undergo diagnostic angiography or coronary revascularization.

CAD in hemodialysis patients is complex due to the calcified nature of the arteries and the extent of coronary lesions.

## Ethical approval

NA.

## Consent

Informed consent was waived due to the study’s retrospective design, based on our institutional policies for de-identified case series.

## Sources of funding

No funding was received for this work.

## Author contribution

N.E.O.: project administration; N.I.: conceptualization and supervision; B.A.: data collection, data analysis; M.B.: writing – original draft; M.E.A.: review and editing.

## Conflicts of interest disclosure

The authors have no competing interests to declare that are relevant to the content of this article.

## Research registration unique identifying number (UIN)

1. Name of the registry: NA.
2. Unique Identifying number or registration ID: NA.
3. Hyperlink to your specific registration (must be publicly accessible and will be checked): NA.

## Guarantor

Mohammed Boutaybi.

## Provenance and peer review

Not commissioned, externally peer-reviewed.
